# Selective JAK2 inhibition by TG101209 reprograms macrophage polarization and alleviates acute lung injury

**DOI:** 10.3389/fimmu.2026.1755208

**Published:** 2026-03-13

**Authors:** Jinxian Ye, Youguang Pan, Canchao Jia, Sijia Liu, Xiang Li, Lixin Zhao, Jiandong Zhang, Xiying Chen, Bingyu Zheng, Zhiyu Chen, Juan Yang, Zhongxiao Lin, Ao Shen, Xin Yang, Lu Liang

**Affiliations:** 1 The Fifth Affiliated Hospital, Key Laboratory of Molecular Target and Clinical Pharmacology, NMPA, and the State Key Laboratory of Respiratory Disease, The School of Pharmaceutical Sciences, Guangzhou Medical University, Guangzhou, China; 2 Department of Thoracic Surgery, Guangdong Provincial People’s Hospital (Guangdong Academy of Medical Sciences), Southern Medical University, Guangzhou, China; 3 State Key Laboratory of New Targets Discovery and Drug Development for Major Diseases, Gannan Innovation and Translational Medicine Research Institute, Gannan Medical University, Ganzhou, China

**Keywords:** acute lung injury (ALI), inflammation, JAK2/STAT3 signaling, macrophage polarization, TG101209

## Abstract

**Background:**

Acute lung injury (ALI) represents a critical respiratory syndrome involving extensive alveolar injury and uncontrolled inflammation, yet it continues to exhibit high mortality rates in the absence of effective treatments. Here we evaluate TG101209, a selective Janus kinase 2 (JAK2) inhibitor, as a modulator of macrophage polarization and a candidate intervention for ALI.

**Methods:**

This study employed both in vivo and in vitro models to investigate the protective effects of TG101209 against ALI. Using lipopolysaccharide (LPS)-induced ALI mice and RAW264.7 inflammatory injury models, the JAK2/STAT3 signaling axis was validated by western blotting and immunofluorescence.

**Results:**

TG101209 alleviated pulmonary inflammation, improved lung function, inhibited M1 polarization, and promoted M2 polarization. Specifically, TG101209 downregulated CD80 and iNOS while upregulating CD163 and Arg1 at both mRNA and protein levels. TG101209 treatment markedly decreased the phosphorylation levels of JAK2 and STAT3 at Ser727 and Tyr705.

**Conclusion:**

TG101209 promotes macrophage polarization toward the M2 phenotype by blocking JAK2/STAT3 activation, indicating its therapeutic value in ALI.

## 1 Introduction

Acute lung injury (ALI), also known as acute respiratory distress syndrome (ARDS), represents a critical clinical condition characterized by severe respiratory insufficiency, impaired oxygen exchange, and extensive pulmonary inflammation accompanied by edema. Despite advances in supportive treatment, the mortality rate remains unacceptably high (1–3). The underlying mechanisms of ALI are multifaceted, involving interactions among immune cells, inflammatory mediators, endothelial barrier disruption, and epithelial cell injury (4). Both direct causes, such as pulmonary infections, and indirect factors, including sepsis, can initiate an overwhelming inflammatory cascade. This response is marked by neutrophil accumulation, elevated secretion of pro-inflammatory cytokines such as TNF-α, IL-1β, and IL-6, and excessive production of reactive oxygen species, which collectively damage the alveolar–capillary interface and contribute to widespread tissue injury (5). These pathological processes are further aggravated by microvascular coagulation and defective repair responses, often progressing into a fibroproliferative stage that leads to irreversible lung remodeling and poor clinical prognosis (4, 5). Although significant progress has been made in elucidating disease mechanisms, effective pharmacological interventions remain lacking, highlighting the pressing need for novel therapeutic strategies for ALI (6).

Macrophages are key regulators of the immune response in ALI, primarily through the secretion of cytokines and chemokines that control the recruitment of immune cells and the amplification of local inflammation (7). Their functional state is highly dependent on environmental cues, enabling them to adopt distinct polarization patterns. Among these, the M1 phenotype drives pro-inflammatory responses, whereas the M2 phenotype promotes anti-inflammatory and reparative processes, exerting contrasting influences on disease outcomes (8). Aberrant polarization of macrophages has been identified as a major contributor to the immunopathology of ALI and other inflammatory disorders, suggesting that strategies aimed at redirecting macrophage polarization may hold therapeutic potential (9).

Recent evidence indicates that the JAK2/STAT3 signaling cascade plays a crucial role in the development of ALI, as its activation has been shown to drive excessive inflammatory reactions and promote macrophage polarization toward the M1 phenotype (10, 11). Conversely, JAK2 inhibition has been reported to suppress M1 polarization and enhance M2-associated anti-inflammatory effects, thereby alleviating lung injury (12–15). This evidence suggests that targeting JAK2-mediated macrophage polarization could offer a new therapeutic approach for ALI.

TG101209, a selective JAK2 inhibitor, has been widely investigated in cancer research for its potent anti-proliferative effects (16–18). However, its potential protective role in ALI and the underlying mechanisms remain largely unexplored. Given the essential role of JAK2/STAT3 signaling and macrophage polarization in the progression of ALI, this study was designed to investigate the therapeutic potential of TG101209 and to clarify its underlying molecular mechanisms using both *in vivo* and *in vitro* models.

## 2 Results

### 2.1 TG101209 attenuates LPS-induced ALI in mice

**Figure 1:**
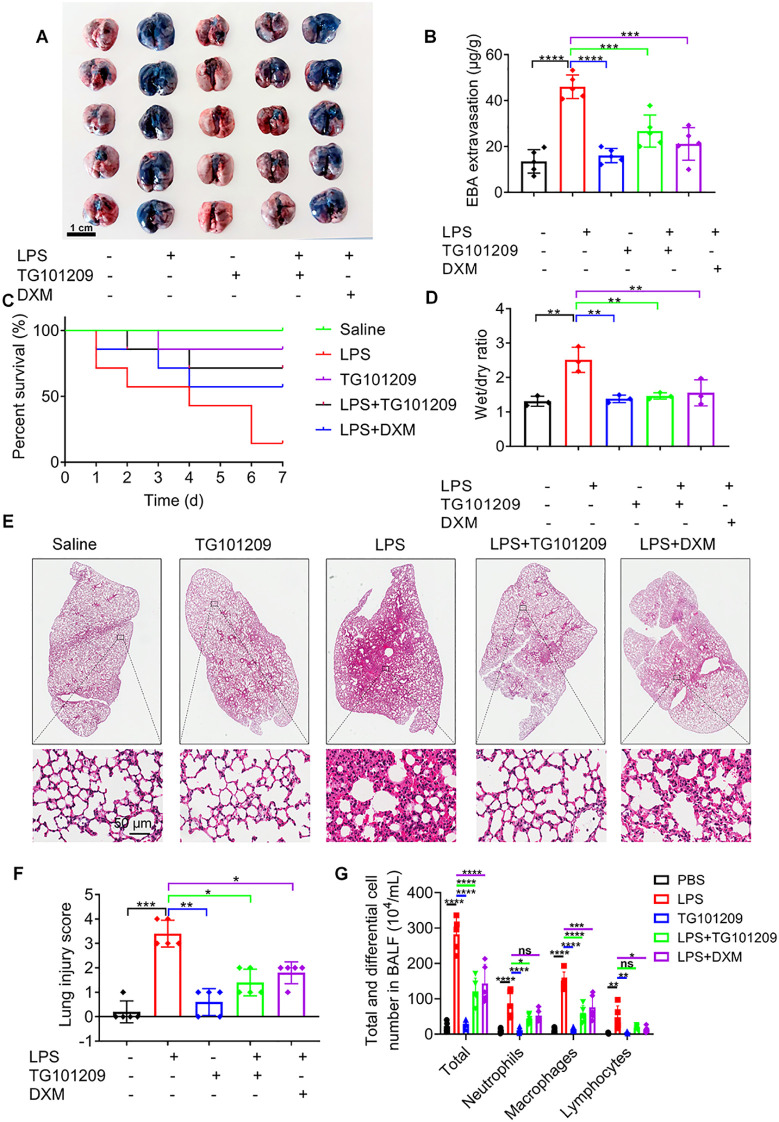
TG101209 alleviates LPS-triggered ALI. **(A)** Representative images showing the distribution of Evans blue dye in lung tissues from mice subjected to different treatments, indicating pulmonary vascular permeability. Evans blue dye was intravenously injected prior to sacrifice. Scale bar = 1 cm. **(B)** Quantitative analysis of Evans blue extravasation in lung tissues, measured by absorbance at 620 nm after formamide extraction. **(C)** Kaplan–Meier survival curves of mice monitored for 7 days following LPS challenge. Mice were treated as indicated, and survival was recorded daily. **(D)** Lung wet-to-dry (W/D) weight ratios used to assess pulmonary edema. Lung tissues were weighed immediately after collection (wet weight) and again after drying at 60 °C for 48 h. **(E)** Representative histological images of lung sections stained with H&E to evaluate lung tissue injury, including alveolar structure disruption, inflammatory cell infiltration, and interstitial edema. Scale bar = 50 μm. **(F)** Quantification of lung injury scores based on H&E-stained sections using a standardized scoring system evaluating alveolar congestion, hemorrhage, inflammatory cell infiltration, and alveolar wall thickness. **(G)** BALF cell counts, including total cells, neutrophils, macrophages, and lymphocytes, determined using a hemocytometer followed by Giemsa staining. For each bar, *n* = 5 mice per group. For the survival curve, *n* = 7 mice per group. Data are presented as mean ± SD. ^*^P < 0.05, ^**^P < 0.01, ^***^P < 0.001, ^****^P < 0.0001 *vs* LPS group.

Multiple *in vivo* assays were conducted to assess the therapeutic role of TG101209 against LPS- triggered ALI. Evans blue staining revealed extensive vascular leakage in the lungs of LPS-treated mice, indicated by intense blue coloration. Treatment with TG101209 markedly reduced the Evans blue signal, approaching levels observed in control mice, suggesting improved vascular integrity (**Figures 1A, B**). Survival analysis further demonstrated that TG101209 significantly increased ALI mouse survival in comparison with the LPS group, and notably, its effect was superior to that of dexamethasone (DXM) (**Figure 1C**). Consistent with these findings, the lung wet-to-dry (W/D) weight ratio, which was elevated in ALI mice, was restored to near-normal levels following TG101209 treatment (**Figure 1D**). Histological analysis using H&E staining showed that LPS induced substantial pathological changes, including neutrophil infiltration, thickened alveolar walls, deposition of hyaline membranes, loss of cellular integrity and irregular cell morphology. TG101209 treatment markedly alleviated these abnormalities, reducing inflammatory cell infiltration and normalizing alveolar structure (**Figure 1E**). Quantitative analysis of lung injury scores confirmed the significant protective effect of TG101209 (**Figure 1F**). As shown in **Figures 1G**, LPS administration induced pronounced lung inflammation, as indicated by elevated numbers of neutrophils, macrophages, and lymphocytes in BALF, whereas treatment with TG101209 significantly attenuated this inflammatory cell infiltration. Additionally, pulmonary function testing using the Buxco system demonstrated that LPS impaired multiple respiratory parameters, including tidal volume, minute volume, peak expiratory flow, FEV_100_ (forced expiratory volume for the first 100 milliseconds), FEV_100_/FVC (forced vital capacity) and lung resistance. TG101209 treatment effectively reversed these functional deficits (**Supplementary Figure 1**). Importantly, no significant histopathological abnormalities were detected in the liver, heart, spleen, or kidney by H&E staining (**Supplementary Figure 2**), indicating a favorable safety profile at the therapeutic dose. Collectively, these results indicate that TG101209 significantly alleviates LPS-induced pathological and functional lung injury in mice.

### 2.2 TG101209 promotes macrophage reprogramming from M1 to M2 phenotype

**Figure 2:**
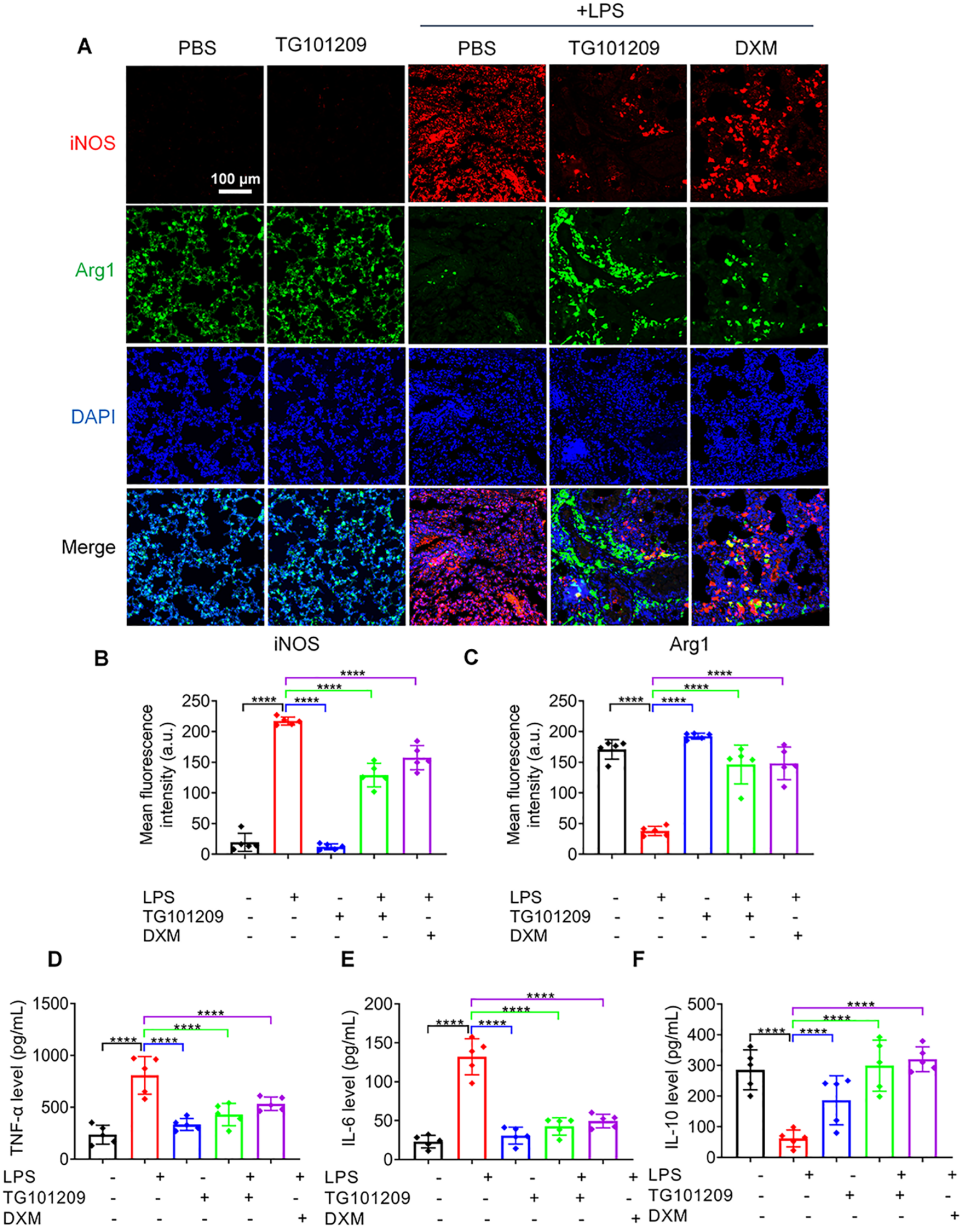
Immunofluorescence staining of pulmonary tissues and inflammatory cytokine levels in LPS-induced ALI. **(A)** Representative immunofluorescence images of lung tissue sections showing M1 macrophages labeled with iNOS (red) and M2 macrophages labeled with Arg1 (green); nuclei were counterstained with DAPI (blue). Lung tissues were collected 72 h after intratracheal LPS administration. Scale bar = 100 μm. **(B, C)** Quantitative analysis of iNOS **(B)** and Arg1 **(C)** fluorescence intensity in lung sections. Fluorescence intensity was quantified using ImageJ software. **(D–F)** Levels of TNF-α **(D)**, IL-6 **(E)**, and IL-10 **(F)** in BALF, measured by ELISA according to the manufacturer’s instructions. For each bar, *n* = 5 mice per group. Data are presented as mean ± SD. ^****^P < 0.0001 *vs* LPS group.

Macrophages are critical regulators of the immune-inflammatory process and serve as key mediators in ALI pathogenesis. We examined the impact of TG101209 on macrophage polarization and inflammatory cytokine production. LPS stimulation prominently enhanced iNOS expression in M1 macrophages while suppressing the M2 marker Arg1, as revealed by immunofluorescence (**Figures 2A–C**). Correspondingly, pro-inflammatory cytokines TNF-α and IL-6 rose significantly, accompanied by a decrease in the anti-inflammatory cytokine IL-10 (**Figures 2D–F**).

**Figure 3:**
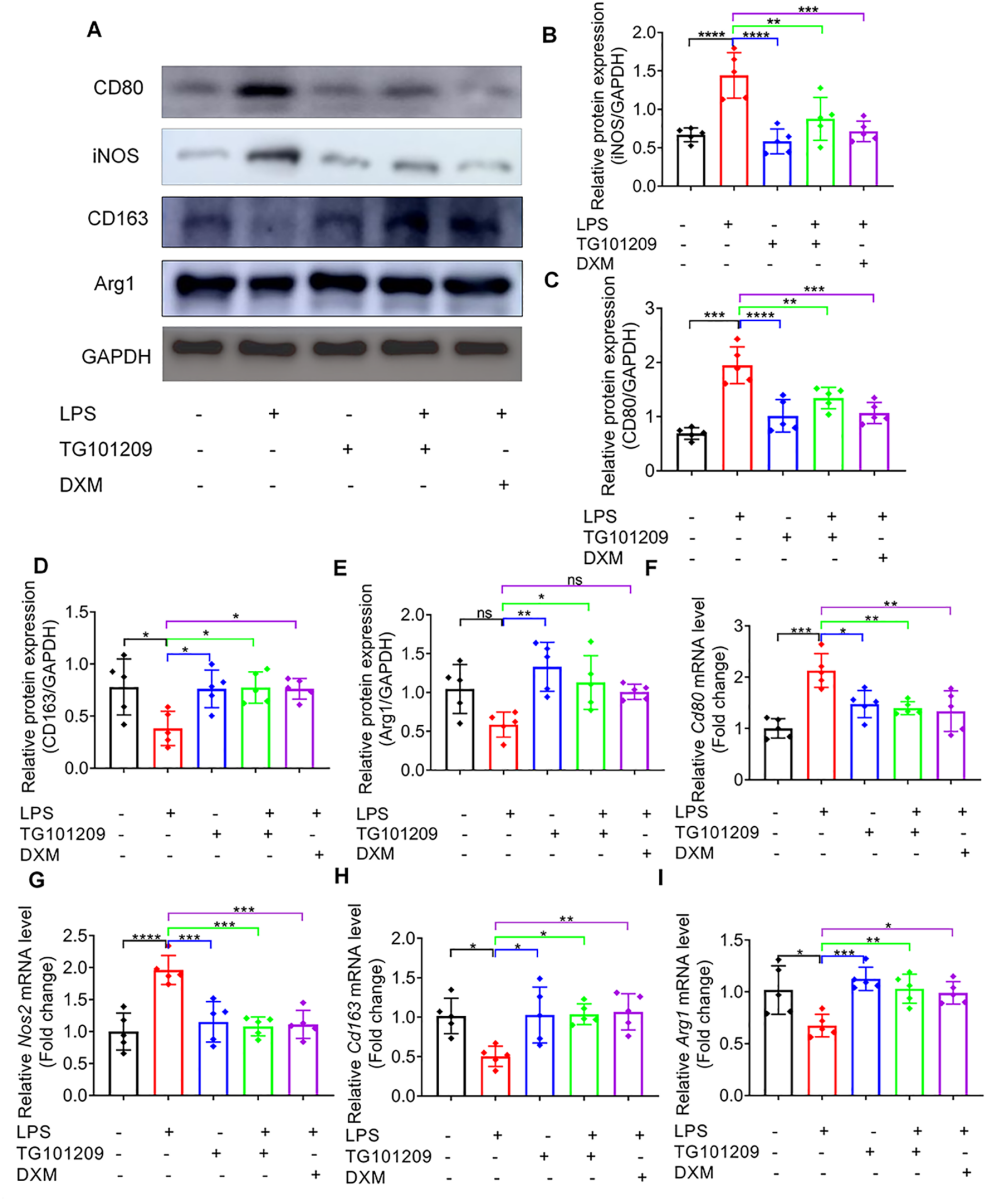
TG101209 regulates protein and mRNA expression of macrophage polarization markers *in vivo*. **(A)** Representative Western blot images of lung tissues showing M1 macrophage markers CD80 and iNOS, and M2 macrophage markers CD163 and Arg1. Lung tissues were collected 72 h after intratracheal LPS administration. GAPDH was used as the loading control. **(B-E)** Quantification of protein levels for CD80 **(B)**, iNOS **(C)**, CD163 **(D)**, and Arg1 **(E)**. Band intensities were measured using ImageJ software and normalized to GAPDH. **(F-I)** Quantitative RT-qPCR analysis of mRNA expression levels of *Cd80***(F)**, *Nos2***(G)**, *Cd163***(H)**, and *Arg1***(I)** in lung tissues. Total RNA was extracted from lung tissue, reverse-transcribed to cDNA, and amplified using gene-specific primers. Expression levels were normalized to *Gapdh*. For each bar, *n* = 5 mice per group. Data are presented as mean ± SD. ^*^P < 0.05, ^**^P < 0.01, ^***^P < 0.001, ^****^P < 0.0001 *vs* LPS group.

Western blotting corroborated these results, showing that LPS markedly elevated the protein expression of M1 markers CD80 and iNOS, whereas TG101209 treatment significantly suppressed these increases (**Figures 3A–C**). Conversely, M2 markers CD163 and Arg1, which were suppressed by LPS, were restored upon TG101209 treatment. The extent of macrophage reprogramming induced by TG101209 was comparable to that observed with dexamethasone (DXM) (**Figures 3A, D, E**). Consistent with protein data, RT-qPCR results demonstrated that TG101209 decreased *Cd80* and *Nos2* mRNA expression while restoring *Cd163* and *Arg1* transcription to levels similar to the control group (**Figures 3F–I**).

**Figure 4:**
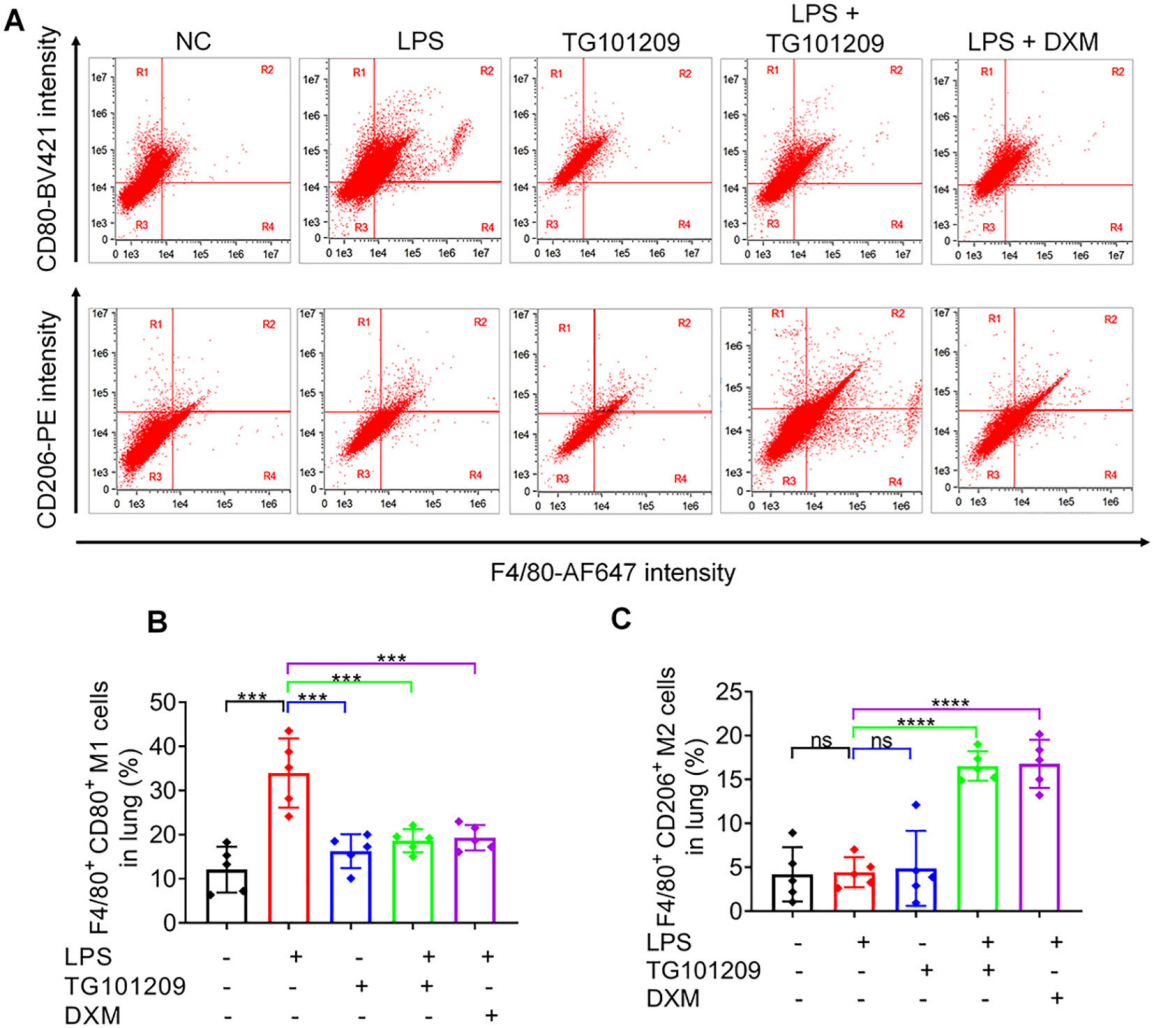
The evaluation of M1 and M2 macrophage subsets in lung tissues by flow cytometry following TG101209 treatment. **(A)** Representative flow cytometry plots showing the distribution of M1 and M2 macrophage subsets in lung tissues from different experimental groups. M1 macrophages were identified as F4/80^+^CD80^+^ cells, and M2 macrophages were identified as F4/80^+^CD206^+^ cells. **(B, C)** Quantitative analysis of the percentages of M1 and M2 macrophages, respectively, among total lung macrophages. For each bar, *n* = 5 mice per group. Data are presented as mean ± SD. ^***^P < 0.001, ^****^P < 0.0001 *vs* LPS group.

Flow cytometric assessment demonstrated a marked increase in the proportion of M1 macrophages after LPS exposure, which was notably attenuated by TG101209 treatment (**Figures 4A, B**). Conversely, the M2 macrophage fraction, which was decreased by LPS, was significantly increased by TG101209 (**Figures 4A, C**).

Overall, these findings suggest that TG101209 alleviates LPS-induced ALI through macrophage reprogramming toward an M2 phenotype, thereby suppressing inflammatory responses and facilitating pulmonary tissue recovery.

### 2.3 TG101209 regulates macrophage polarization *in vitro*

*In vitro* experiments with RAW264.7 macrophages were performed to clarify the underlying mechanism of TG101209. The CCK-8 assay demonstrated that TG101209 exhibited no significant cytotoxicity at concentrations below 5 μM within 24 hours (**Supplementary Figure 3**). Therefore, 1 μM was selected as a safe and effective concentration for subsequent mechanistic studies.

**Figure 5:**
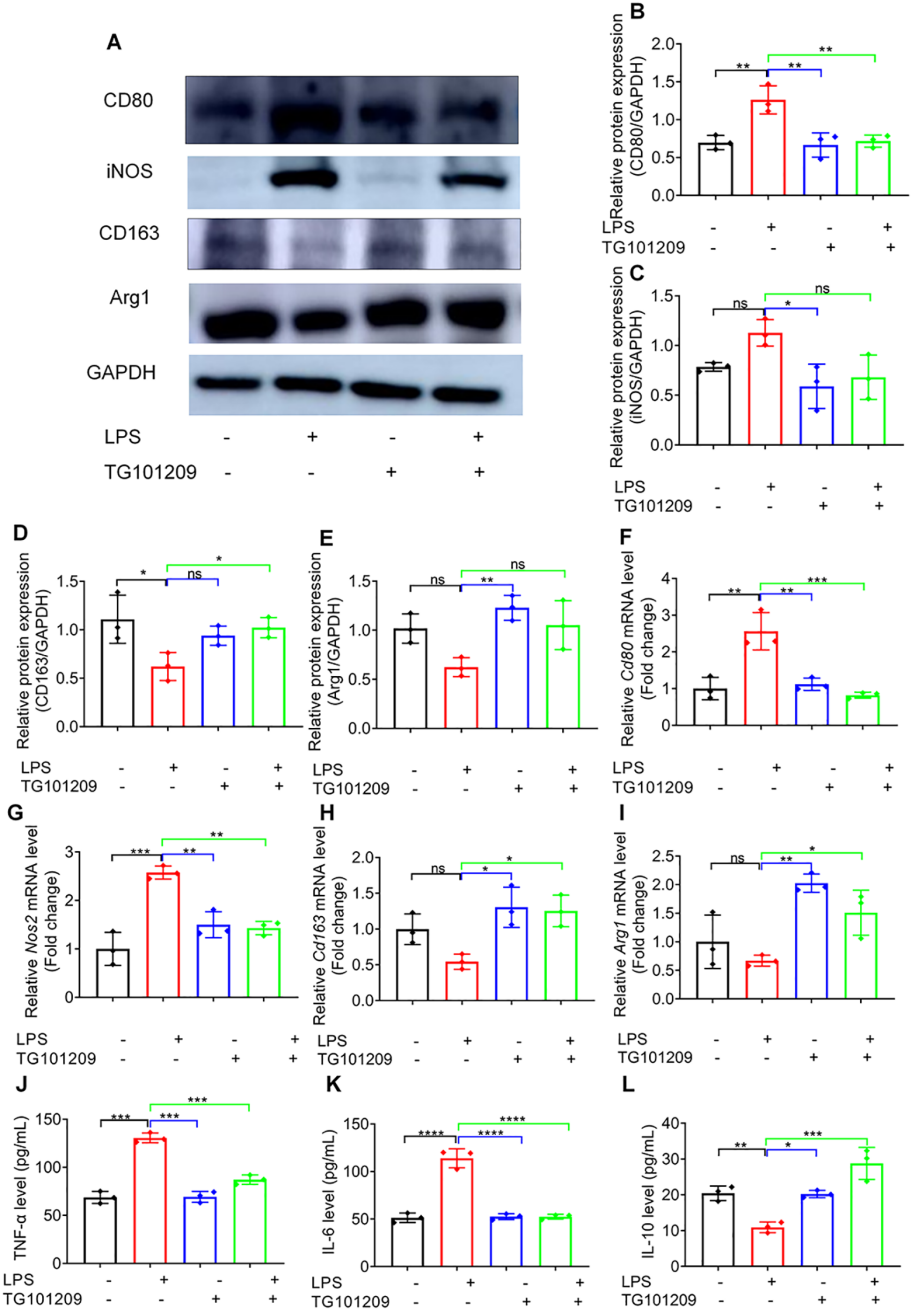
TG101209 regulates macrophage polarization *in vitro*. **(A)** Representative Western blot images showing the protein expression of CD80, CD163, iNOS, and Arg1 in RAW264.7 cells under different treatment conditions. **(B–E)** Quantitative analysis of CD80, CD163, iNOS, and Arg1 protein levels, normalized to GAPDH and expressed relative to the control group. **(F–I)** Relative mRNA expression levels of *Arg1*, *Cd80*, *Cd163*, and *Nos2* in RAW264.7 cells, as determined by RT-qPCR and normalized to *Gapdh*. **(J–L)** Concentrations of TNF-α, IL-6, and IL-10 in the culture supernatant, measured by ELISA. For *in vitro* cell experiments, *n* = 3 independent replicates per group. Data are presented as mean ± SD. ^*^P < 0.05, ^**^P < 0.01, ^***^P < 0.001, ^****^P < 0.0001 *vs* LPS group.

In line with the *in vivo* observations, exposure to LPS strongly enhanced the protein expression of M1-related markers such as CD80 and iNOS, while simultaneously downregulating M2-associated proteins CD163 and Arg1. Administration of TG101209 effectively counteracted these effects, leading to a marked reduction of CD80 and iNOS expression and a recovery of CD163 and Arg1 levels toward normal conditions (**Figures 5A–E**).

At the transcriptional level, RT-qPCR analysis demonstrated that LPS significantly elevated the mRNA expression of the M1 genes *Cd80* and *Nos2*, whereas TG101209 treatment notably suppressed this induction. Conversely, the LPS-mediated decrease in *Cd163* and *Arg1* mRNA expression was substantially reversed following TG101209 exposure (**Figures 5F–I**), indicating its role in driving macrophages toward an anti-inflammatory phenotype.

Furthermore, TG101209 markedly reduced LPS-stimulated TNF-α and IL-6 production and restored IL-10 in culture supernatants (**Figures 5J–L**). Collectively, these findings demonstrate that TG101209 exerts potent immunomodulatory effects by promoting macrophage polarization from the M1 to the M2 phenotype.

### 2.4 TG101209 attenuates ALI by blocking JAK2/STAT3 signaling

**Figure 6:**
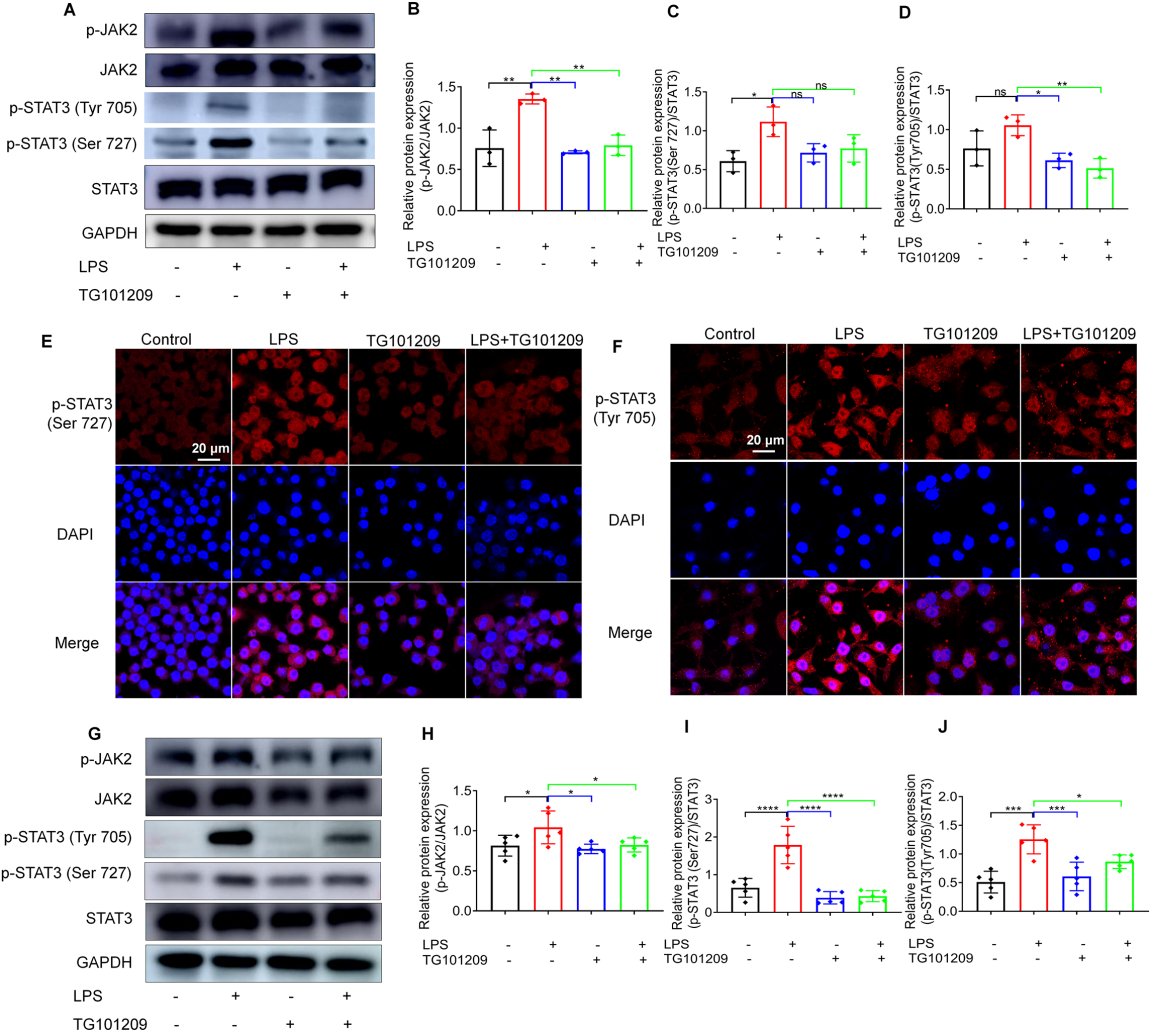
TG101209 attenuates ALI by blocking JAK2/STAT3 signaling. **(A)** Representative Western blot images showing the protein expression levels of total JAK2, phosphorylated JAK2 (p-JAK2), total STAT3, and phosphorylated STAT3 at Ser727 and Tyr705 in RAW264.7 cells under the indicated treatment conditions. **(B-D)** Quantitative analysis of the phosphorylation ratios of p-JAK2/JAK2 and p-STAT3 (Ser727)/STAT3 and p-STAT3 (Tyr705)/STAT3, normalized to total protein levels and expressed relative to the control group. **(E)** Representative immunofluorescence images showing p-STAT3 (Ser727, red) localization with DAPI-stained nuclei (blue). Scale bar = 20 μm. **(F)** Representative immunofluorescence images showing p-STAT3 (Tyr705, red) localization with DAPI-stained nuclei (blue). Scale bar = 20 μm. **(G)** Representative Western blot images showing the protein expression levels of total JAK2, p-JAK2, total STAT3, and phosphorylated STAT3 at Ser727 and Tyr705 in lung tissues isolated from mice in the indicated treatment groups. **(H-J)** Quantitative analysis of the phosphorylation ratios of p-JAK2/JAK2, p-STAT3 (Ser727)/STAT3, and p-STAT3 (Tyr705)/STAT3 in mouse lung tissues, normalized to total protein levels and expressed relative to the control group. For *in vitro* cell experiments, *n* = 3 independent replicates per group. For *in vivo* experiments, *n* = 5 mice per group. Data are presented as mean ± SD. ^*^P < 0.05, ^**^P < 0.01, ^***^P < 0.001, ^****^P < 0.0001 *vs* LPS group.

To further elucidate the molecular mechanism of TG101209, we assessed the activity of the JAK2/STAT3 signaling pathway. Phosphorylation of JAK2 and STAT3 at Ser727 and Tyr705 was evaluated using western blot analysis. As shown in **Figures 6A–D**, LPS exposure significantly elevated the p-JAK2/JAK2 and p-STAT3/STAT3 ratios at Ser727 and Tyr705 sites relative to controls. Administration of TG101209 markedly reduced these phosphorylation levels.

In addition, immunofluorescence staining was used to visualize the intracellular localization of phosphorylated STAT3. Consistent with the western blot findings, LPS treatment induced prominent nuclear translocation of p-STAT3 in RAW264.7 cells (**Figures 6E, F**). Notably, TG101209 administration effectively reduced nuclear accumulation of p-STAT3, suggesting that blockade of STAT3 activation occurs.

Importantly, similar inhibitory effects on JAK2/STAT3 phosphorylation were also observed in lung tissues isolated from LPS-challenged mice treated with TG101209, further supporting the *in vivo* relevance of these findings (**Figures 6G–J**). Taken together, these results demonstrate that TG101209 attenuates LPS-induced macrophage activation partially by inhibiting the JAK2/STAT3 signaling cascade, thereby preventing excessive inflammatory responses and contributing to the macrophages reprogramming from M1 to M2 phenotype.

## 3 Discussion

ALI remain life-threatening disorders characterized by pulmonary edema and uncontrolled inflammation, with no effective pharmacological treatment currently available. Increasing evidence suggests that inhibition of the JAK2/STAT3 signaling cascade can mitigate sepsis-induced ALI and that modulation of this pathway alleviates LPS-induced pulmonary injury (19–21). Thus, targeting JAK2 represents a promising therapeutic strategy. TG101209, a small-molecule JAK2 inhibitor widely investigated in oncology (18, 22), has not yet been systematically investigated for its anti-inflammatory potential. However, its potential efficacy against ALI has not been clearly defined. In this work. We hypothesized that TG101209 alleviates ALI, which was subsequently evaluated through *in vivo* and *in vitro* models. Histological examination and Evans Blue extravasation assays revealed that TG101209 effectively reduced lung injury and vascular permeability in mice exposed to LPS. Moreover, TG101209 downregulated the levels of TNF-α and IL-6 in BALF and significantly improved pulmonary function compared with the LPS group, collectively demonstrating its protective role against ALI *in vivo*.

Macrophages are central regulators of immune homeostasis and inflammation, with their polarization status critically influencing disease outcomes (23). Polarizing macrophages away from the M1 pro-inflammatory phenotype toward an M2 anti-inflammatory profile is increasingly recognized as a promising strategy for managing inflammatory disorders (24). In ALI, aberrant M1 polarization drives excessive cytokine release and lung injury, whereas M2 polarization promotes resolution and tissue repair (25, 26). Our study demonstrated that TG101209 ameliorated LPS-induced ALI, at least in part, by suppressing M1 polarization while enhancing M2 polarization. Specifically, TG101209 downregulated M1 markers (CD80, iNOS) and upregulated M2 markers (Arg1, CD163), underscoring its immunoregulatory potential in macrophage reprogramming.

To delineate this mechanism, *in vitro* assays were conducted. We found that TG101209 effectively promoted macrophage transition from the M1 to the M2 phenotype by blocking JAK2/STAT3 activation. These observations align with previous reports that JAK2/STAT3 signaling serves as an inhibitory modulator of M1 polarization and a critical driver of M2 polarization (27). As a potent JAK2 inhibitor, TG101209 significantly suppressed JAK2 activity, leading to impaired JAK2/STAT3 signaling and, consequently, attenuation of LPS-induced inflammatory injury. Consistent with earlier studies, inhibition of JAK2/STAT3 activation is associated with the resolution of inflammation (28), preventing tissue damage caused by uncontrolled M1 activation and facilitating a shift toward M2 polarization (29–31).

Although independent human RNA-seq or single-cell datasets were not generated in the present study, the translational relevance of our findings is supported by published genome-wide transcriptomic analyses in patients with ALI/ARDS. Notably, a comprehensive expression profiling study of peripheral blood from acute-stage versus recovery-stage ARDS patients identified multiple differentially expressed genes associated with inflammatory signaling pathways, including components related to JAK–STAT signaling (32). Among these, IL8, PTGS2 (COX-2), and STAT1 are well-established downstream targets or functional components of the JAK–STAT axis. Activation of the IL-6–JAK2–STAT3 pathway has been shown to directly induce IL8 transcription, thereby promoting neutrophil recruitment and amplifying inflammatory responses (33, 34). PTGS2 is a STAT3-responsive gene widely implicated in inflammation-associated tissue injury across immune and epithelial compartments (35–37). Although STAT1 is classically linked to interferon signaling, it shares upstream activation by JAK kinases and exhibits functional crosstalk with STAT3 during inflammatory lung injury (38, 39). Importantly, these JAK–STAT–associated genes have been consistently identified in human ARDS transcriptomic studies, supporting the clinical relevance of the JAK2/STAT3-related inflammatory programs observed in our experimental models (40).

Taken together, although the present study does not directly dissect the cell-type-specific effects of JAK2 inhibition in non-macrophage lung populations, macrophages serve as key upstream regulators of cytokine production, neutrophil recruitment, and secondary injury to epithelial and endothelial cells in ALI/ARDS. Therefore, targeting macrophage JAK2–STAT3 signaling is expected to indirectly influence inflammatory responses across multiple lung cell compartments (41–43). This limitation has been explicitly acknowledged, and future studies incorporating single-cell transcriptomics, human datasets, or lineage-specific approaches will be required to fully define the broader cellular effects of JAK2 inhibition in ALI/ARDS.

Overall, our findings identify TG101209 as a potent immunomodulatory candidate that attenuates ALI through JAK2/STAT3-dependent macrophage reprogramming, providing a strong mechanistic and translational rationale for further investigation.

## 4 Conclusions

**Figure 7:**
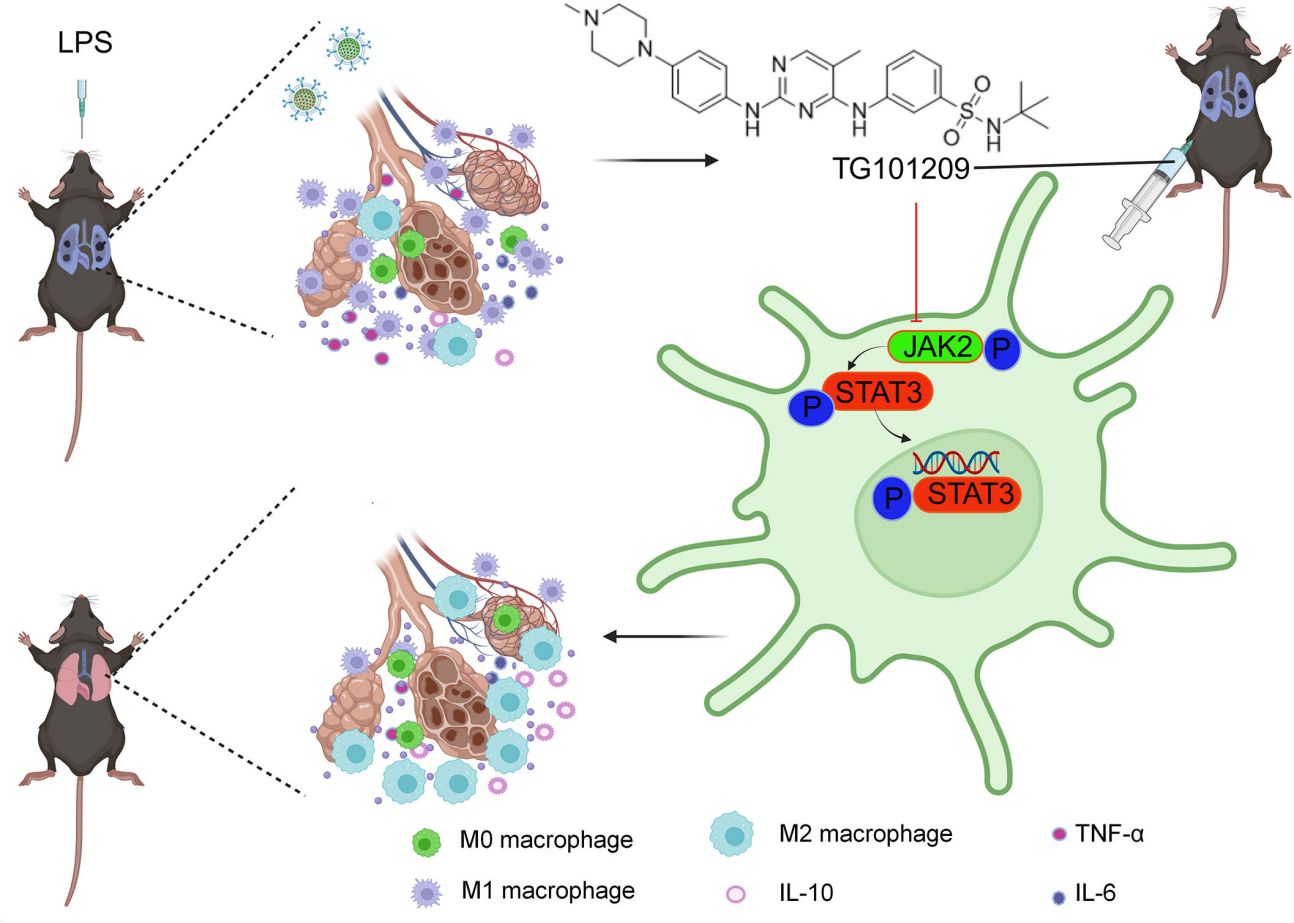
TG101209 alleviates LPS-mediated lung injury and facilitates the macrophage polarization shift from the M1 state and toward an M2 phenotype by suppressing JAK2/STAT3 signaling.

This study provides evidence that TG101209 alleviates LPS-triggered ALI through suppression of the JAK2/STAT3 signaling cascade, which in turn facilitates the macrophage polarization shift from the M1 pro-inflammatory phenotype toward an M2 anti-inflammatory profile. These findings suggest that TG101209 functions as a potent immunomodulatory agent, capable of restoring immune homeostasis and alleviating excessive inflammatory responses in ALI (**Figure 7**).

## 5 Materials and methods

### 5.1 Materials

TG101209, LPS from *Escherichia coli* O55:B5 and DXM were obtained from MedChem Express (Shanghai, China). Fetal bovine serum (FBS), Dulbecco’s Modified Eagle’s Medium (DMEM), and penicillin/streptomycin were provided by Gibco BRL/Life Technologies (Grand Island, NY, USA). Hematoxylin and eosin (H&E), phosphate-buffered saline (PBS), and 4% paraformaldehyde were obtained from Servicebio (Wuhan, China). Anti-STAT3 (1:1000, 86 kDa, Selleck), anti-p-STAT3 (Ser727) (1:1000, 86 kDa, Selleck), anti-p-STAT3 (Tyr705) (1:1000, 86 kDa, Selleck), anti-CD80 (1:1000, 60 kDa, Abcam), anti-CD163 (1:1000, 150 kDa, Abcam), anti-Arg1 (1:1000, 35 kDa, Abcam), anti-iNOS (1:1000, 131 kDa, Abcam), anti-p-JAK2 (1:1000, 130 kDa, Huabio), anti-JAK2 (1:1000, 125 kDa, Selleck), anti-Tubulin (1:1000, 57 kDa, Abcam) and anti-GAPDH (1:2000, 36 kDa, Abcam) were purchased from Affinity (Melbourne, Australia). Evo M-MLV RT Premix for q-PCR was from Accurate Biology (Hunan, China). PerfectStayt^®^ Green qPCR SuperMix was obtained from TransGen Biotech (Beijing, China), and the radioimmunoprecipitation assay (RIPA) buffer was acquired from Solarbio (Beijing, China). Phenylmethanesulfonyl fluoride (PMSF) and loading buffer were sourced from Beibokit (Shanghai, China).

### 5.2 Cell culture and stimulation

RAW 264.7 macrophages were procured from the Cell Resource Center of Peking Union Medical College (Beijing, China) and cultured in complete medium at 37 °C with 5% CO_2_. For inflammatory activation, cells were exposed to LPS (50 ng/mL, 24 h) with or without TG101209 at indicated concentrations.

### 5.3 Cell viability assay

The potential cytotoxicity of TG101209 was assessed using both live/dead cell staining and CCK-8 assay. For qualitative evaluation, live and dead cells were distinguished by Calcein-AM and Propidium Iodide (PI) staining (Beyotime, Beijing, China), and the fluorescence signals were subsequently captured with a confocal laser scanning microscope (CLSM). For quantitative evaluation, the relative viability was calculated as the ratio of treated to control optical density.

### 5.4 ELISA assay

Cell culture supernatants and BALF were analyzed by ELISA to quantify IL−10, IL−6, and TNF−α, following kit instructions to ensure technical consistency.

### 5.5 RNA extraction and RT-qPCR

RNA was isolated from either cells or lung tissues using TRIzol reagent. Subsequently, 500 ng of the extracted RNA was reverse-transcribed into complementary DNA (cDNA) using the Evo M-MLV RT kit. The generated cDNA was then diluted, and 1 μL was employed as a template in SYBR Green to quantify the levels of the target genes. The primers used are listed in **Supplementary Table 1**.

### 5.6 Western blotting assay

Proteins were isolated, measured, and immobilized on PVDF membranes. After blocking, membranes were incubated with primary antibodies specific to the proteins of interest, followed by secondary antibodies. Bands were visualized using the SuperSignal chemiluminescent substrate (Thermo Fisher Scientific, Waltham, MA, USA) and imaged. Quantification of relative protein expression was performed using Image J, with normalization to housekeeping controls.

### 5.7 Immunofluorescence assay

For immunofluorescence analysis, both RAW264.7 macrophages and mouse lung tissue sections were prepared and incubated overnight at 4 °C with primary antibodies against Arg1 (1:150; Abcam), iNOS (1:150; Abcam), and phospho-STAT3 at Ser727 and Tyr705 (1:200; Selleck). After thorough PBS washes, the samples were incubated in the dark with appropriate fluorophore-conjugated secondary antibodies. Fluorescent signals in both cells and tissue sections were captured using a ZEISS LSM880 confocal laser scanning microscope (Germany).

### 5.8 Animal experiments

Male C57BL/6 mice (6–7 weeks old, SPF grade) were obtained from Guangdong Vital River Laboratory Animal Technology Co., Ltd. All animal protocols were approved by the Institutional Animal Care and Use Committee of Guangzhou Medical University (GY2023-713).

To establish the acute lung injury (ALI) model, mice were administered lipopolysaccharide (LPS) intratracheally at a dose of 2 mg/kg in a total volume of 50 μL sterile PBS. Animals were randomly assigned to five experimental groups (n = 5 per group): (1) Control group, intratracheally administered saline; (2) LPS group, intratracheally administered LPS (2 mg/kg); (3) TG101209 group, treated with TG101209 alone (40 mg/kg); (4) LPS + TG101209 group, co-treated with LPS (2 mg/kg) and TG101209 (40 mg/kg); (5) LPS + DXM group, serving as a positive control, co-treated with LPS (2 mg/kg) and dexamethasone (DXM, 5 mg/kg). Two hours after intratracheal administration of saline or LPS, mice in the corresponding groups were intratracheally treated with TG101209 or DXM as indicated.

After 72 hours of LPS challenge, pulmonary function was assessed using the Buxco pulmonary function testing system (Data Sciences International, DSI, DE, USA) in accordance with the manufacturer’s instructions and previously published protocols (44). Briefly, mice were randomly placed in a whole-body plethysmography chamber connected to a highly sensitive pressure transducer to detect subtle pressure changes within the chamber. Pulmonary function parameters, including tidal volume, minute volume, peak expiratory flow, forced expiratory volume in the first 100 ms (FEV100), FEV100/forced vital capacity (FVC), and lung resistance, were recorded and analyzed.

Subsequently, the mice were deeply anesthetized with pentobarbital sodium (50 mg/kg, i.p.), and euthanized by cervical dislocation prior to tissue collection. Lung tissues and BALF were harvested for subsequent analyses. For survival studies, a separate set of mice received LPS intratracheally, followed by the indicated treatments, and survival was monitored every 24 hours for 7 consecutive days.

### 5.9 BALF collection and cell counting

Briefly, each mouse lung was lavaged three times with 0.6 mL of sterile saline. Total BALF cells were collected and counted using a hemocytometer. Subsequently, the cells were subjected to Giemsa staining for differential cell counting under a light microscope, allowing identification and quantification of neutrophils, macrophages, and lymphocytes.

### 5.10 Wet/dry weight ratio of lung tissue

The middle lobe of the left lung was dissected and weighed to obtain the wet weight. Samples were then dried at 45 °C until a stable dry weight was achieved, and the wet-to-dry (W/D) ratio was calculated to evaluate the extent of pulmonary edema.

### 5.11 EBA extravasation assessment

Pulmonary vascular permeability was evaluated using Evans Blue (EB) dye. 40 min prior to lung tissue collection, mice were administered EB via intravenous injection into the orbital vein. After euthanasia, lung tissues were excised, weighed, and incubated in formamide at room temperature for 72 hours to extract the dye. The amount of extravasated EB was then quantified using a UV-visible spectrophotometer.

### 5.12 Histological analysis

The tissue samples were collected, fixed in 10% formalin, embedded in paraffin, sectioned at 4 μm, and stained with H&E for blinded semi−quantitative scoring of injury. The severity of lung damage was assessed by scoring the area percentage of pulmonary edema, alveolar interstitial inflammation and hemorrhage, and atelectasis observed in each microscopic field of H&E stained tissue sections. The severity of lung injury was graded using a five-point scale: 0, no observable damage; 1, involvement of ≤25% of the field; 2, ≤50% affected; 3, ≤75% affected; and 4, diffuse injury throughout the field (45, 46). All assessments were independently performed by two blinded pathologists to ensure unbiased evaluation.

### 5.13 Flow cytometry analysis of pulmonary macrophages

After euthanasia, mouse lung tissues were harvested and cut into small pieces. The tissues were digested in a buffer containing 1 mg/mL Collagenase D and 0.1 mg/mL DNase I (both from Roche, Indianapolis, IN, USA) in Hanks’ balanced salt solution, and mechanically dissociated using a GentleMACS Dissociator (Miltenyi Biotec) according to the manufacturer’s instructions. The resulting homogenates were filtered through a 40-μm nylon mesh to obtain single-cell suspensions. Residual red blood cells were lysed using red blood cell lysis buffer.

The single-cell suspensions were then subjected to immunofluorescent staining with fluorophore-conjugated antibodies, including FITC-conjugated anti-mouse CD80, APC-conjugated anti-mouse F4/80, and PerCP-Cy5.5-conjugated anti-mouse CD206 (all from BD Biosciences, CA, USA). Stained cells were analyzed using an ImageStreamx Mark II flow cytometer (Merck, Darmstadt, Germany). Macrophages expressing both F4/80 and CD80 were classified as M1 phenotype, whereas those expressing F4/80 and CD206 were classified as M2 phenotype.

### 5.14 Statistical analysis

All experiments were performed using independent, parallel group designs. Data were tested for normality using Shapiro-Wilk normality test. Normally distributed data are presented as mean ± standard deviation (SD). Statistical significance among multiple groups was assessed using one-way analysis of variance (ANOVA) followed by Tukey’s *post hoc* test. A *p* value < 0.05 was considered statistically significant. Statistical analyses were performed using GraphPad Prism 8.0.

## Data Availability

The raw data supporting the conclusions of this article will be made available by the authors, without undue reservation.
