# Supplementary material for: Selective JAK2 inhibition by TG101209 reprograms macrophage polarization and alleviates acute lung injury
Source: Front Immunol. 2026 Mar 13;17:1755208. doi: 10.3389/fimmu.2026.1755208 (PMC13021449; doi:10.3389/fimmu.2026.1755208)
Supplement: Supplementary file 1 [file DataSheet1.pdf]

Supporting information

Selective JAK2 Inhibition by TG101209 Reprograms Macrophage Polarization and Alleviates Acute Lung Injury

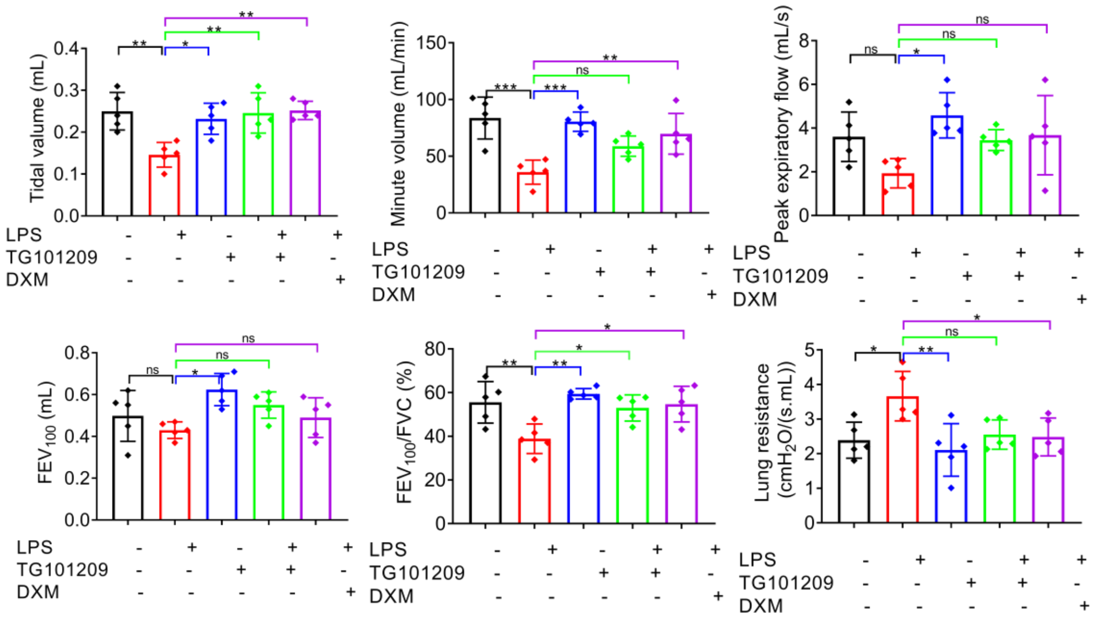

Figure S1. TG101209 improves pulmonary function in LPS-induced ALI mice. Pulmonary function was assessed 72 h after LPS administration using the Buxco pulmonary function testing system. Parameters measured included tidal volume, minute volume, peak expiratory flow, forced expiratory volume in the first 100 ms (FEV100), the ratio of FEV100 to forced vital capacity (FEV100/FVC), and lung resistance. For each bar, n = 5 mice per group. Data are presented as mean ± SD. $^{*}P < 0.05$, $^{**}P < 0.01$, $^{***}P < 0.001$, $^{****}P < 0.0001$ vs LPS group.

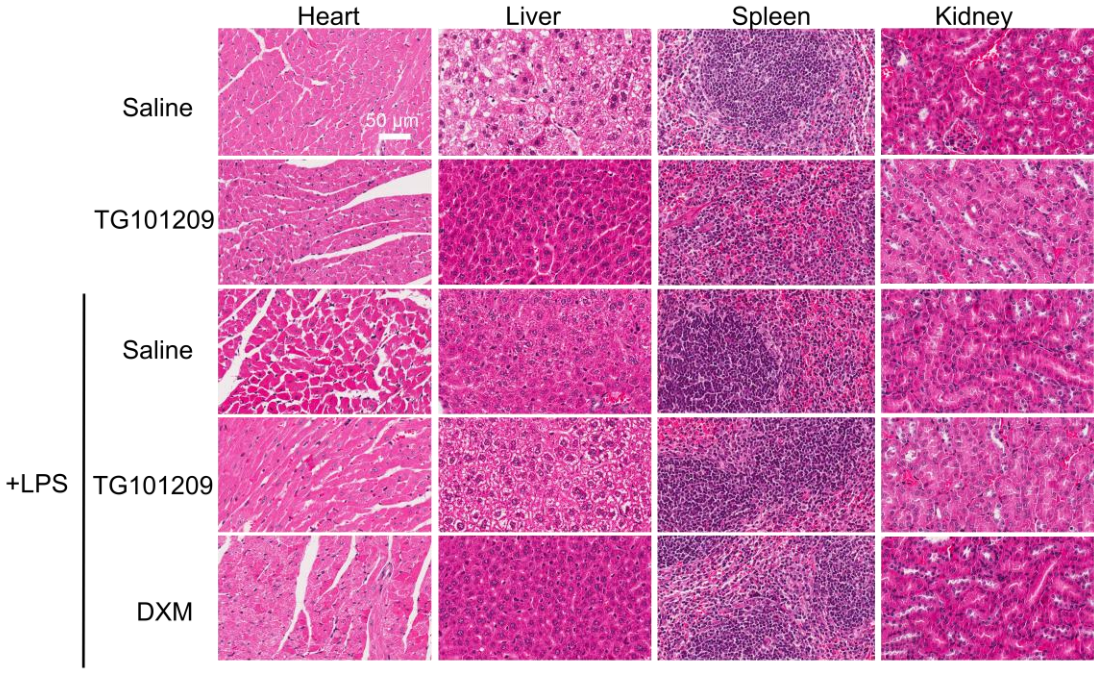

Figure S2. HE staining of major organs. Representative histological images of the heart, liver, spleen, lung, and kidney were obtained from mice after the indicated treatments to assess potential tissue toxicity or pathological alterations. Scale bar = 50 μm.

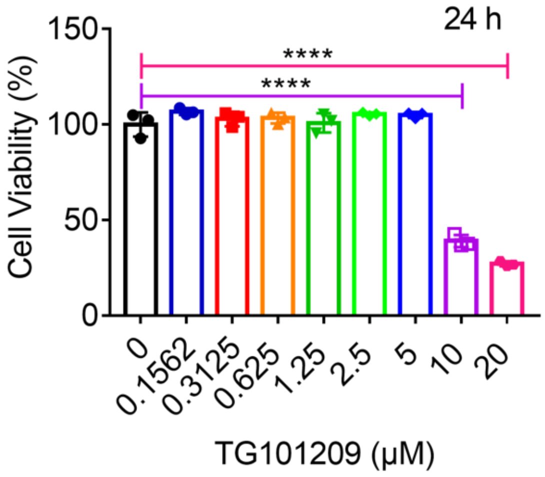

Figure S3. Cytotoxicity of TG101209 in RAW264.7 cells evaluated by CCK-8 assay. Cells were treated with increasing concentrations of TG101209 for 24 h, followed by incubation with CCK-8 reagent. Cell viability was quantified by measuring absorbance

at 450 nm and normalized to the untreated control group. For *in vitro* cell experiments, n = 3 independent replicates per group. Data are presented as mean ± SD. $^{****}P < 0.0001$ vs untreated group.

Table S1 Primers for RT-qPCR.

Gene	Forward/ Reverse	Sequence (5'-3')
Arg1	Forward	CTCCAAGCCAAAGTCCTTAGAG
	Reverse	AGGAGCTGTCATTAGGGACATC
Nos2	Forward	GTTCTCAGCCCAACAATACAAGA
	Reverse	GTGGACGGGTCGATGTCAC
Cd80	Forward	GCAGGATACACCACTCCTCAA
	Reverse	AAAGACGAATCAGCAGCACAA
Cd163	Forward	TGGAGTTGCCCTTTCTACCC
	Reverse	GCCACTTGCTCTGAAGGACAT
Il-6	Forward	TAGTCCTTCCTACCCCAATTTCC
	Reverse	TTGGTCCTTAGCCACTCCTTC
Tnf-a	Forward	GCCACCTTTTGACAGTGATGAG
	Reverse	ACGGGAAAGACACAGGTAGC
Il-10	Forward	CTTACTGACTGGCATGAGGATCA
	Reverse	GCAGCTCTAGGAGCATGTGG
Gapdh	Forward	CCCTCAACGACCACTTTGTC

	Reverse	AGGGGAGATTCAGTGTGGTG
Jak2	Forward	GGAATGGCCTGCCTTACAATG
	Reverse	TGGCTCTATCTGCTTCACAGAAT
Stat3	Forward	AGAACCTCCAGGACGACTTTG
	Reverse	TCACAATGCTTCTCCGCATCT
